# Echogenicity of Dupuytren’s nodules is correlated to myofibroblast load and nodule hardness

**DOI:** 10.1177/17531934211050214

**Published:** 2021-10-07

**Authors:** Sanne Molenkamp, Whangzao Song, Marjolein Bloembergen, Dieuwke C. Broekstra, Paul M. N. Werker

**Affiliations:** 1 Department of Plastic Surgery, University of Groningen, Groningen, The Netherlands; 2 Department of Pathology, University of Groningen, Groningen, The Netherlands

**Keywords:** Dupuytren’s contracture, ultrasonography, tonometry, myofibroblasts

## Abstract

This study aimed to determine the association between the echogenicity of Dupuytren’s disease nodules and myofibroblast load, and between echogenicity and nodule hardness. Thirty-eight nodules were assessed sonographically. The echogenicity of nodules was measured objectively with Image J (grey-value) and subjectively by visual inspection (hypo-, mixed and hyper-echogenicity). These findings were compared with myofibroblast load measured by histopathological analysis. In a different cohort, 97 nodules were assessed for grey-value and nodule hardness using a tonometer. There was a moderate, significant, negative association between grey-value and myofibroblast load and the subjective visual measurements corresponded to this finding. There was also a moderate, significant, negative association between grey-value and nodule hardness. Ultrasound and tonometry may be useful in the selection of patients for possible future preventive treatments.

## Introduction

One of the unresolved problems in the management of Dupuytren’s disease is the prevention of progression of early disease into contractures that need surgical treatment (Nanchahal et al., 2018; van Rijssen et al., 2012; Yin et al., 2017). No drug has yet been found that can prevent progression of the disease (Werker and Degreef, 2018). Although Dupuytren’s disease has a high prevalence in Northern and Western Europe, only a small percentage of patients ultimately have surgical treatment for the disease (Degreef and De Smet., 2010; Gudmundsson et al., 2000; Lanting et al., 2013). Should an effective preventive treatment be developed, the next step would be to identify patients with mild symptoms, who are at risk of developing progressive disease. Currently there is no way to do this.

Activity can be linked to the cellularity of nodules based on the different pathological stages described by Luck (1959). Nodules progress from the highly cellular proliferative stage, to the involutional stage, in which cells align to lines of tension, to the residual stage, where they mainly consist of acellular fibrous tissue (Lam et al., 2010; Luck, 1959). It is therefore more likely that nodules that are in the proliferative stage (active nodules) will show progression than nodules that are already in the residual phase. A small case-series has shown that MRI signal intensity corresponds to the cellularity of the Dupuytren’s tissue microscopically (Yacoe et al., 1993). Creteur et al. (2010) suggested that the echogenicity (the ‘greyness’ of Dupuytren’s nodules examined by ultrasound) may also correspond to cellularity. They hypothesized that hypo-echogenic nodules have high cellularity and hyper- to iso-echogenic nodules have low cellularity. As yet, no histopathological study has been done to substantiate this hypothesis and no clinical study has confirmed that these ‘active’ nodules, with high cellularity, are more likely to progress.

Nodules vary in tissue hardness depending on the different histopathological stages of Dupuytren’s disease. This may be another measure of disease activity. Tonometry can distinguish Dupuytren’s tissue from normal tissue and its reliability is excellent (Ball et al., 2017). However, there is no information about nodule hardness and its possible relation with disease stage.

Previous studies have shown that myofibroblasts are instrumental for contracture formation in Dupuytren disease (Gabbiani and Majno, 1972; Verjee et al., 2009). If ultrasound and MRI findings correspond not only to the cellularity of nodules, but to myofibroblast load in particular, this would be helpful in defining the activity of nodules. Since ultrasound is readily available and has low costs, it would be preferable to MRI to assess the activity of Dupuytren’s nodules. In situations where ultrasound is not available, tonometry might be another way to measure nodule activity.

In this study our primary aim was to assess the association between echogenicity of Dupuytren’s nodules and myofibroblast load. We also analysed the association between the echogenicity of nodules and their hardness.

## Methods

### Ultrasound and myofibroblast load

Patients with Dupuytren’s disease undergoing limited fasciectomy were asked to participate. Rays that were operated previously were excluded, because of the possibility of scar tissue interfering with sonographic and histological findings. This study was presented to the local ethics committee, which decided that formal approval was unnecessary (2017.560). All participants gave written informed consent.

**Figure 1.**
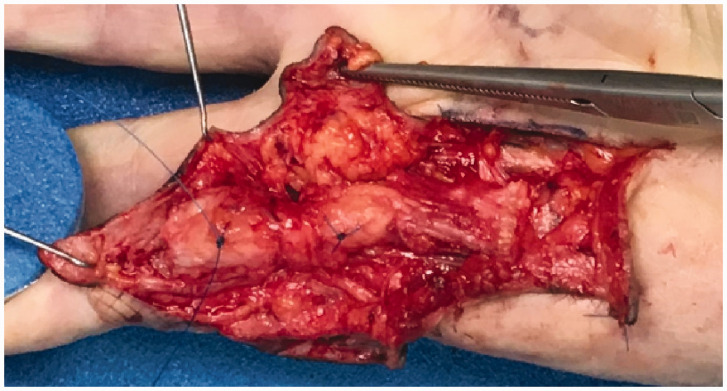
Two nodules marked with stitches.

**Figure 2.**
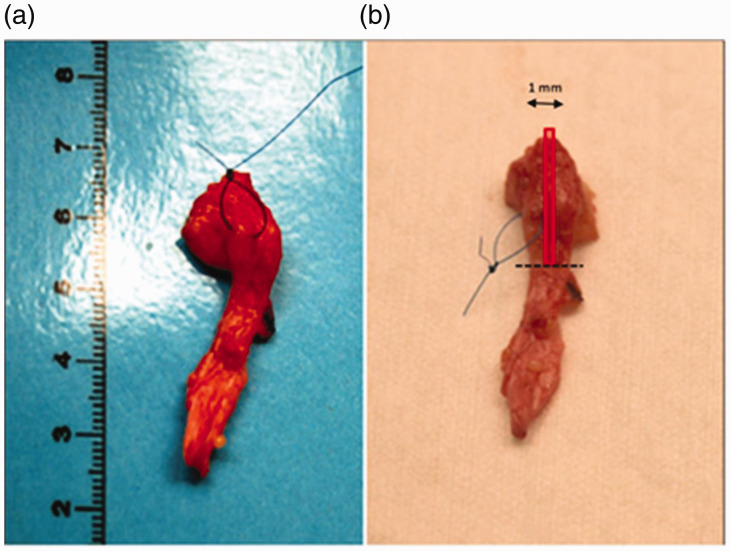
(a) Example of a removed cord. (b) After fixation with formalin, the nodule is cut from the cord (dotted black line) and then a 1-mm thick section is cut from the centre of the nodule, in the sagittal plane (red rectangle).

Patients that agreed to participate were examined before surgery. Dupuytren’s nodules within the affected rays of the hand undergoing surgery were marked on the skin with a water-resistant skin marker. A nodule was defined as a clinically palpable subcutaneous mass within a Dupuytren’s cord. If possible, the affected rays with the least contracture were selected, because in our experience a severe contracture may interfere with ultrasound imaging and the assessment of greyness. Subsequently, sonographic images were obtained in the sagittal plane of the same area that was marked on the skin. We used a mobile ultrasound device (Esaote Mylab one, Genova, Italy) with an 18 MHz linear transducer. During surgery, after elevating the skin flaps from the cord, but before excising the cord itself, a stitch was placed at the centre of the nodule corresponding to the mark on the skin (Figure 1). The cord was then removed and fixed in 4% buffered formalin. After 3–5 days the nodules were excised from the cords with the same length as measured during ultrasound. In the centre of the nodules a 1-mm thick section was taken in the sagittal plane (Figure 2). This was subsequently embedded in paraffin. Sections 2–4 µm thick were cut from the sample and stained with haematoxylin and eosin (H&E). A commercially available smooth muscle actin (SMA) antibody (clone 1A4, 1:400) was used to identify myofibroblasts (DAKO, Glostrup, Denmark). Immunohistochemistry staining in the Benchmark Ultra automated immunostainer (Ventana, Tuscon, AZ) was optimized using an amplification step (Ultraview detection system). Antigen retrieval was not applied. Myofibroblast load determined by immunohistochemistry was compared with results obtained by H&E staining. All pathological specimens were reviewed by an expert pathologist (AJHS, see Acknowledgement section), specialized in the diagnostic pathology of soft tissue tumours.

The study was done in accordance with the code of conduct for responsible use of human tissue that is used in the Netherlands (Dutch Federation of Biomedical Scientific Societies; http://www.federa.org).

The two primary outcome measures were echogenicity and myofibroblast load. Echogenicity was measured subjectively by the primary researcher (SM) while carrying out the ultrasound. The nodules were scored as either hypo-echogenic (darker), mixed echogenicity or hyper-echogenic (lighter), when comparing them to the underlying flexor tendons. Echogenicity was measured objectively by calculating the grey-value of a nodule using Image J (Abramoff et al., 2004). The border of the measured Dupuytren’s tissue was defined by drawing a thin line around the tissue. Subsequently Image J was used to calculate the mean grey-value within that border. Grey-value can range from 0–255. The lower the value, the darker the nodule.

Histolopathological myofibroblast load was measured by digitizing the stained histological sections and using Image J to measure the red colour of the myofibroblasts. The percentage of red was then calculated by dividing the area of red by the total area of the tissue, with higher percentages indicating more myofibroblasts (i.e. higher myofibroblast load). This procedure was done by a researcher at the department of pathology (WS), also using Image J (Abramoff et al., 2004).

The researchers made their measurements blinded from each other, to reduce the risk of bias.

### Ultrasound and nodule hardness

Measurement of nodule hardness was done using a tonometer in an existing longitudinal cohort in which the natural course of Dupuytren’s disease has been studied (Lanting et al., 2016). This study was approved by the institutional review board (2011.397). All participants gave informed consent.

In the patients that agreed to undergo an extra measurement of tissue hardness during their regular follow-up visits, a palmar nodule was selected in a ray with no history of previous surgery, preferably isolated and not yet part of a cord. Sonographic images were obtained in the transverse and sagittal plane. Subsequently, the tissue hardness of the same nodule was measured using a tonometer (type RX-1600-OO durometer; Rex Gauge Company Inc., IL, USA). Hardness was measured in the centre of the nodule, while balancing the tonometer on its tip in a vertical position. The placement of the tip was marked to enable five repeated tonometry measurements.The nodule hardness was calculated by using the mean of the five tonometry measurements.

The two primary outcome measures were echogenicity (grey-value) and nodule hardness.

The mean grey-value for the different planes was calculated in the same way as described above. The mean grey-value of the two planes was used as outcome measure.

### Statistical analysis

Patient and nodule characteristics were described by means and standard deviations or by medians and interquartile ranges (in continuous variables) and by frequencies and percentages (in categorical variables).

The widths and depths of the Dupuytren’s samples were normally distributed for both the ultrasound images and the histology images. Comparison of means between these groups was therefore performed with a paired samples *t*-test.

**Figure 3.**
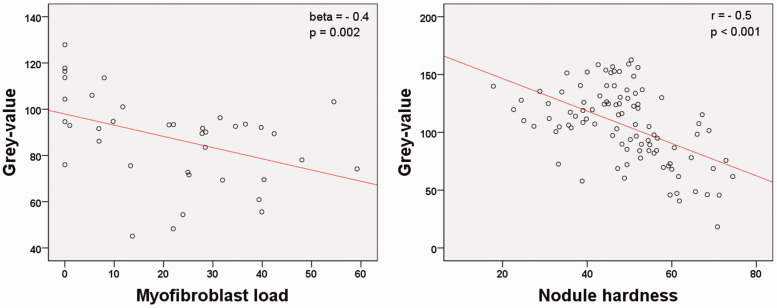
(a) Scatterplot indicating a linear relation between grey-value and myofibroblast load. (b) Scatterplot indicating a linear relation between grey-value and nodule hardness.

In both cohorts the two outcome measures showed a linear relation (Figure 3). Since multiple nodules from the same patient were included in the comparison of histopathology and US, we used a general linear mixed model to assess the strength of the association. Myofibroblast load was included as the outcome, and US grey-value as the fixed effect (robust estimation, a technique to gain model estimators that are less sensitive to violations of the model assumptions than non-robust estimators). A random intercept was included for each patient, to account for the fact that multiple nodules per patient were included. Degrees of freedom were calculated using the Satterthwaite approximation. The association between grey-value and nodule hardness was calculated using Pearson’s *r*, since a single observation was done per patient. The analyses were done in SPSS version 23 and an alpha of 5% was used.

## Results

### Ultrasound and myofibroblast load

Eighteen patients with primary Dupuytren’s disease agreed to participate, of whom ten were male. The mean age was 66 (SD 6.8) years. Thirty-eight Dupuytren’s nodules were surgically removed from these patients after having been studied by ultrasound. The median preoperative total passive extension deficit (TPED) was 30° (interquartile range 60°–10°) in the rays from which the nodules were obtained. Seventeen nodules were obtained from the little finger and 13 from the ring finger. The remaining nodules were obtained the thumb (two), index finger (one) and middle finger (five).

The mean grey-value at ultrasound was 87.4 and the mean myofibroblast load at histology was 22%. The mean lengths of the Dupuytren’s samples on the pathology images were significantly shorter than on the ultrasound images (*p* = 0.009). The mean widths of the samples were comparable. The mean measurements of the samples are shown in Table 1.

**Table 1.** Mean measurements of Dupuytren nodules.

	Ultrasound Mean (SD)	Pathology Mean (SD)	*p*-value
Grey-value	87.4 (19.5)		
Myofibroblast load, %		22 (17)	
Length, mm	13.5 (4)	12.1 (2.5)	**0.009**
Width, mm	4.7 (1.6)	4.9 (1.6)	0.321

Significant values shown in bold font.

**Figure 4.**
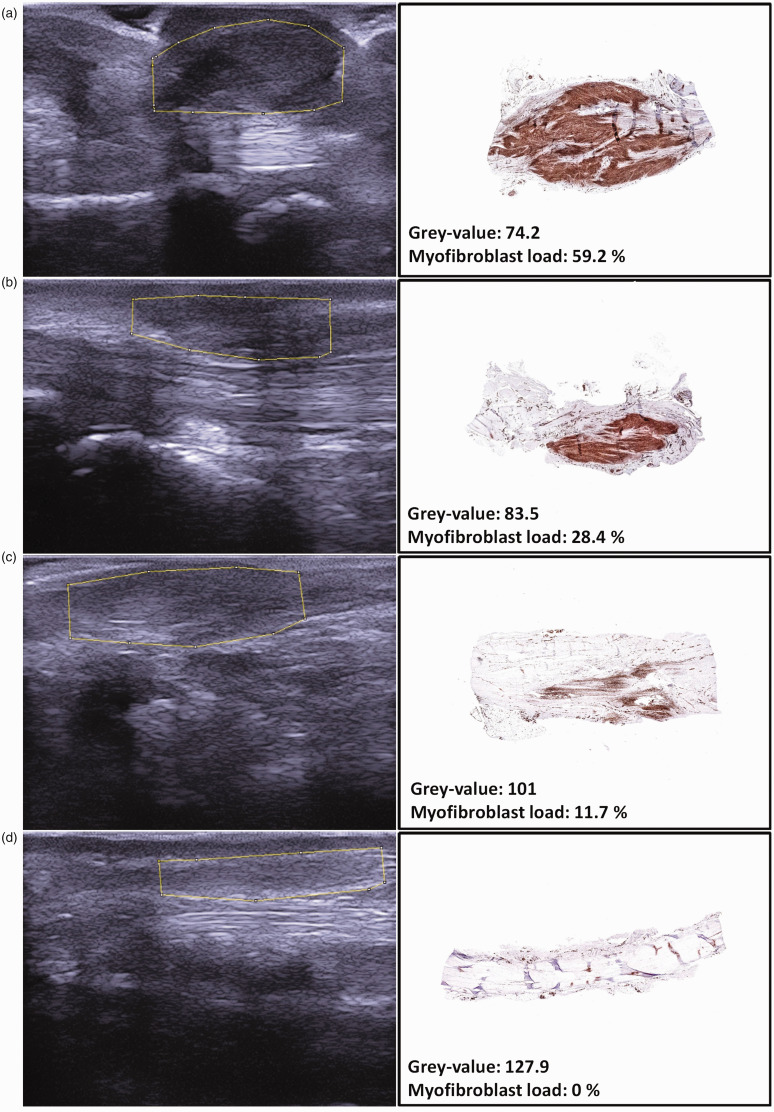
Example of nodules with different grey-values and myofibroblast loads. Subjectively, nodules become lighter on ultrasound from image (a) (darkest) to (d) (lightest), which is objectified by an increasing mean grey-value, measured with Image J. As the grey-value increases, the myofibroblast load decreases.

There was a negative association between grey-value and myofibroblast load (beta = –0.4; *p* = 0.002). Figure 4 shows examples of the relationship between the ultrasound and histopathological images. When comparing the subjective and objective measurements of echogenicity, hypo-echogenicity of nodules corresponded to the lowest mean grey-value. In nodules with mixed echogenicity, the mean grey-value increased and was highest in hyper-echogenic nodules. Mean myofibroblast load decreased from hypo-, mixed to hyper-echogenic nodules (Table 2).

**Table 2.** Subjective measurements of Dupuytren nodules.

	Mean grey-value (SD)	Mean myofibroblast load % (SD)
Hypo-echogenic (*n* = 21)	74.9 (15.3)	30 (14)
Mixed echogenicity (*n* = 8)	97.7 (8)	23 (17)
Hyper-echogenic (*n* = 9)	107.2 (12.6)	2.4 (3.4)

### Ultrasound and nodule hardness

Ninety-seven patients participating in the ongoing study of the natural course of Dupuytren’s disease had an additional measurement of nodule hardness (Table 3). There was a negative association between the grey-value and nodule hardness (*r* = –0.5; *p* < 0.001).

**Table 3.** Descriptive statistics of grey-value versus nodule hardness.

	*n* = 97	Missing, *n* (%)
Mean age, years (SD)	69.0 (9.2)	0 (0)
Gender, *n* (% female)	32 (33.0)	0 (0)
Contracture present, *n* (%)	21 (21.6)	3 (3.1)
Median TPED in degrees (IQR)^a^	14 (80 to 29)	
Mean sagittal grey value sagittal (SD)	106.8 (33.1)	0 (0)
Mean transverse grey value (SD)	105.3 (33.3)	1 (1)
Mean grey value (SD)^b^	106.1 (32.3)	0 (0)
Mean nodule hardness (SD)	48.8 (12.0)	0 (0)

TPED: total passive extension deficit; IQR: interquartile range.

a Median TPED (IQR) among those having contractures.

b Mean of the sagittal and transverse grey values.

## Discussion

Our study showed that there was a moderate association between the grey-value of Dupuytren’s nodules at ultrasound and the myofibroblast load at histology. Also, the subjective interpretation of grey-value of nodules was consistent with the objective measurements performed by Image J. In both hypo-echogenic (grey-value: 74.9) and in nodules with mixed echogenicity (grey-value: 97.7) evident clusters of myofibroblasts were found. Furthermore, hyper-echogenic nodules had a grey-value of at least 107.2 on ultrasound. Histopathological results corresponded to this finding, since a mean myofibroblast load of only 2.4% was found in these hyper-echogenic nodules. When looking at our ultrasound images side-by-side with the pathology samples, it is clear that the ultrasound image and the histological section of the same area have comparable features (Figure 4). This implies that ultrasound may indeed be used as a diagnostic tool to select nodules that are eligible for possible preventive treatments. However, the association coefficient was moderate, which may be explained by several factors. There may have been some variability between the area that was measured with ultrasound and the actual sample that was cut from the cord. The difference in mean length of the Dupuytren’s tissue on ultrasound and histopathological images may be a reflection of this variation. The difference may also be explained by the tissue undergoing changes after the tension is released on removal of the tissue and during the fixation and dehydration process. Other factors may be observer variation, and the possibility that overall echogenicity varies between patients. In a future study, it may be better to use a relative grey-value (compared with the surrounding tissue) instead of an absolute grey-value.

We also found a moderate association between the grey-value of a nodule and its hardness. The darker the nodule appears on ultrasound, the harder it is and vice versa. This leads to the hypothesis that nodule hardness is also correlated to myofibroblast load. It seems logical that a more active nodule containing more myofibroblasts (dark image) is harder because of its higher cell content and related matrix deposition. A further histopathological study should investigate this hypothesis.

Previous studies that have investigated whether the echogenicity of Dupuytren’s nodules corresponds to cellular activity have used only subjective measures to define the echogenicity (i.e. hypo- , mixed or hyper-echogenic) (Creteur et al., 2010; Molenkamp et al., 2019). In this study we also used an objective continuous variable (the grey-value).

**Figure 5.**
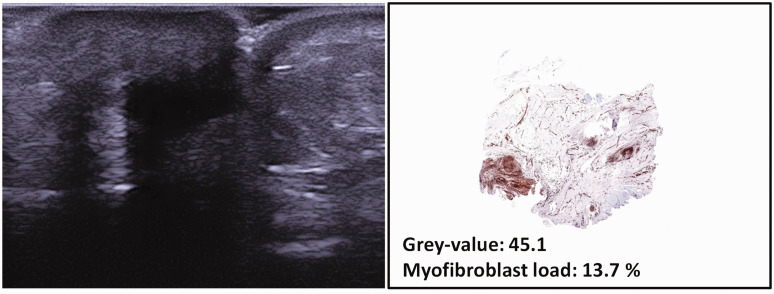
Example of a nodule with a very low grey-value and a low myofibroblast load, which may be caused by artefacts because of severe contracture. Total passive extension deficit was 75°.

A limitation of our study was in the groups we studied. In the cohort that we used for examining the association between ultrasound and myofibroblast load, all the patients had advanced stage Dupuytren’s disease. Ideally, we would have liked to have studied patients with Dupuytrens’ disease but without contractures (Nanchahal et al., 2017). In the cohort that we used for examining the association between ultrasound and nodule hardness, we included such mildly affected patients. It is unethical to remove nodules from these patients, as it is known that more recurrences are seen after removal of active nodules (Balaguer et al., 2009). Histological analysis was therefore impossible. Ultrasound examination is more difficult in patients with contractures. In the presence of severe contractures, the probe cannot always be placed in full contact with the surrounding skin in a sagittal plane, even when sufficient ultrasound gel is used. This may cause artefacts, which may wrongly be interpreted as Dupuytren’s disease areas with a low grey-value. Figure 5 shows an example of a nodule that was wrongly interpreted as being hypoechogenic because of artefacts.

We found that nodule hardness corresponds to ultrasound. It is not possible to say whether tonometry can also identify clusters of myofibroblasts, because no histopathological study was done. A comparison of tonometry and myofibroblast load should be made in further research.

Further research is necessary to investigate whether a dark ultrasound image, a high myofibroblast load and high nodule hardness do indeed correspond to a more aggressive disease course. If a reliable ultrasound classification can be made, this will greatly improve the selection of patients with early Dupuytren’s disease that are at risk of progression. Tonometry of nodules may be an interesting and easy-to-perform additional investigation, but its value has to be studied more thoroughly in different stages of Dupuytren’s disease.
